# Renal Cell Carcinoma Metastasized to Pagetic Bone

**DOI:** 10.7759/cureus.737

**Published:** 2016-08-15

**Authors:** Ashley Ramirez, Bo Liu, Baiywo Rop, Michelle Edison, Michael Valente, Jeremy Burt

**Affiliations:** 1 Diagnostic Radiology, Florida Hospital-Orlando

**Keywords:** pagetic bone, metastatic disease, renal cell carcinoma, abdominal and pelvic ct, paget's disease

## Abstract

Paget’s disease of the bone, historically known as osteitis deformans, is an uncommon disease typically affecting individuals of European descent. Patients with Paget’s disease of the bone are at increased risk for primary bone neoplasms, particularly osteosarcoma. Many cases of metastatic disease to pagetic bone have been reported. However, renal cell carcinoma metastasized to pagetic bone is extremely rare. A 94-year-old male presented to the emergency department complaining of abdominal pain. A computed tomography scan of the abdomen demonstrated a large mass in the right kidney compatible with renal cell carcinoma. The patient was also noted to have Paget’s disease of the pelvic bones and sacrum. Within the pagetic bone of the sacrum, there was an enhancing mass compatible with renal cell carcinoma. A subsequent biopsy of the renal lesion confirmed renal cell carcinoma.

Paget’s disease of the bone places the patient at an increased risk for bone neoplasms. The most commonly reported sites for malignant transformation are the femur, pelvis, and humerus. In cases of malignant transformation, osteosarcoma is the most common diagnosis. Breast, lung, and prostate carcinomas are the most common to metastasize to pagetic bone. Renal cell carcinoma associated with Paget’s disease of the bone is very rare, with only one prior reported case. Malignancy in Paget's disease of the bone is uncommon with metastatic disease to pagetic bone being extremely rare. We report a patient diagnosed with concomitant renal cell carcinoma and metastatic disease within Paget’s disease of the sacrum. Further research is needed to assess the true incidence of renal cell carcinoma associated with pagetic bone.

## Introduction

Paget’s disease of the bone, historically known as osteitis deformans, is an uncommon disease typically affecting individuals of European descent [1-4]. Patients with Paget’s disease of the bone are at increased risk for primary bone neoplasms, particularly osteosarcoma. The rate for sarcomatous degeneration is reported to be between 0.2-1% [3, 5]. We present a rare case of renal cell carcinoma metastasized to pagetic bone. This study was approved by the institutional review board of Florida Hospital. Patient consent was not required for this report.

## Case presentation

A 94-year-old Caucasian male presented to the emergency department with severe abdominal pain. His medical history was significant for hypertension, but the patient was recently taken off his hypertensive medication due to low blood pressure. He had no significant family history. A physical examination proved to be unremarkable other than chronic aphasia resulting from a stroke two years prior. The patient also had reduced ambulation secondary to right foot pain attributed to claudication. A laboratory work-up demonstrated no significant abnormality.

**Figure 1:**
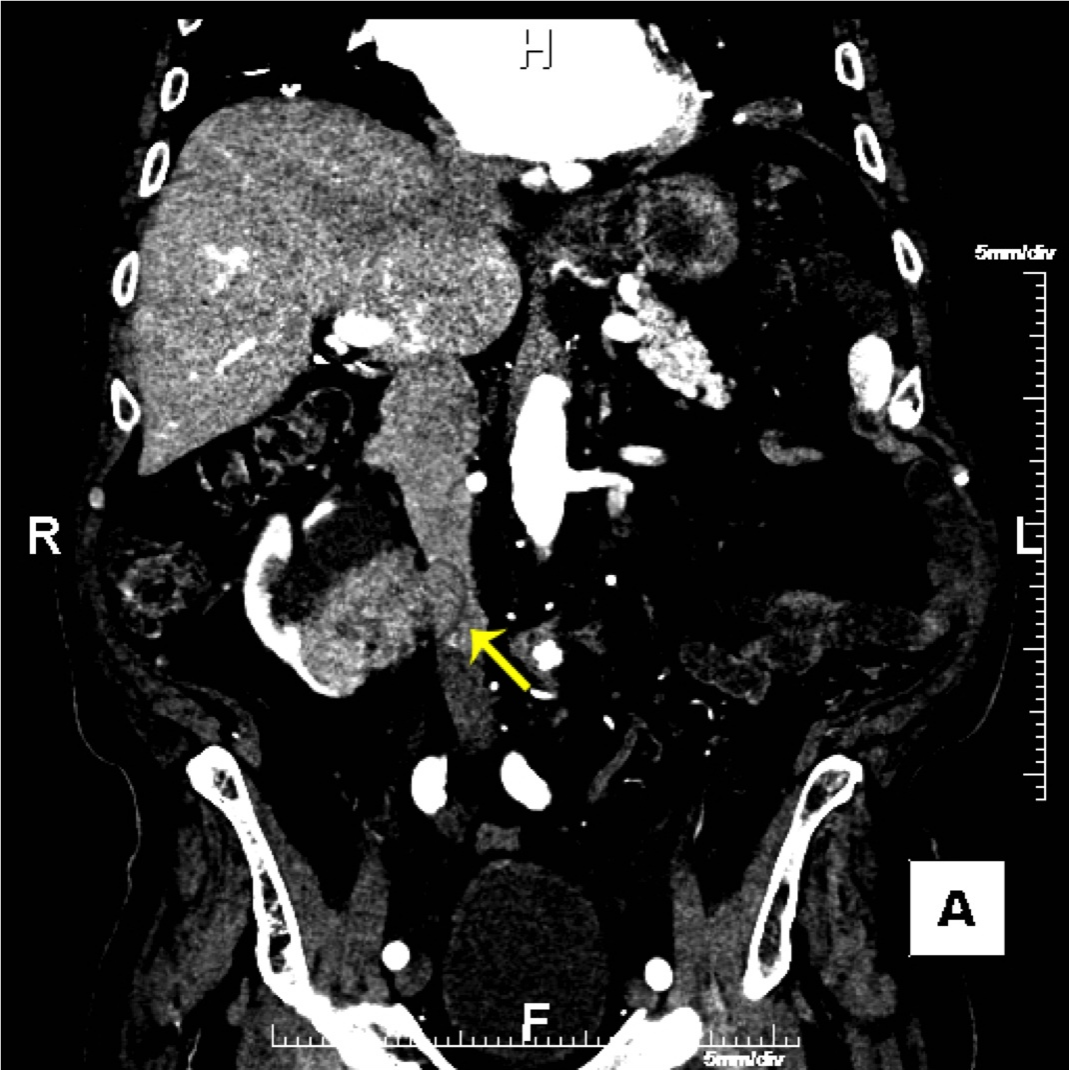
A Contrast-Enhanced CT of the Abdomen and Pelvis Indicating Renal Cell Carcinoma A coronal contrast-enhanced CT of the abdomen and pelvis: renal cell carcinoma of the lower pole of the right kidney with invasion of the accessory right renal vein and inferior vena cava (yellow arrow).

**Figure 2:**
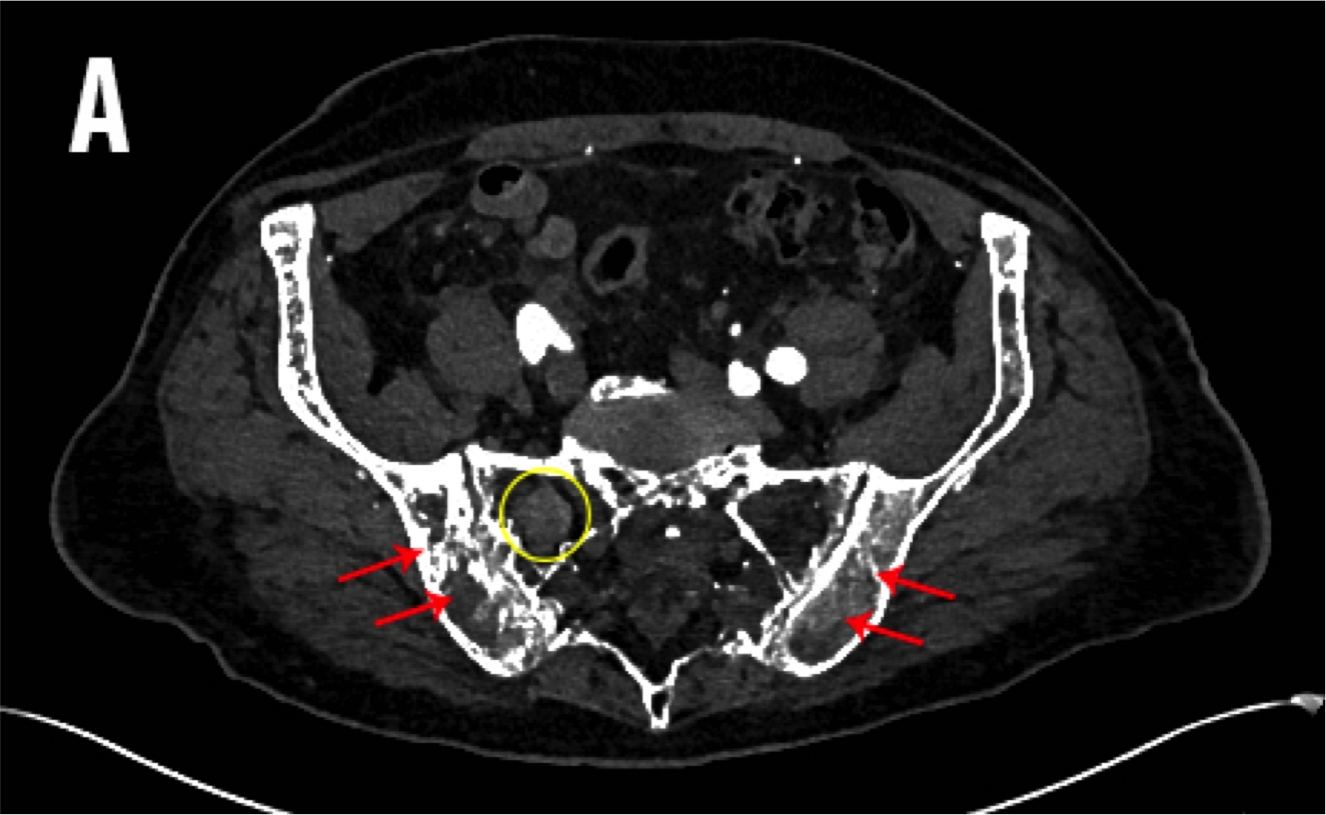
An Axial CT of Pagetic Bone Containing a Metastasis An axial contrast-enhanced CT through the pelvis: characteristic pagetic bone observed within the pelvis and sacrum (red arrows). Also seen is an enhancing mass in the upper right sacrum compatible with metastatic renal cell carcinoma (yellow circle).

**Figure 3:**
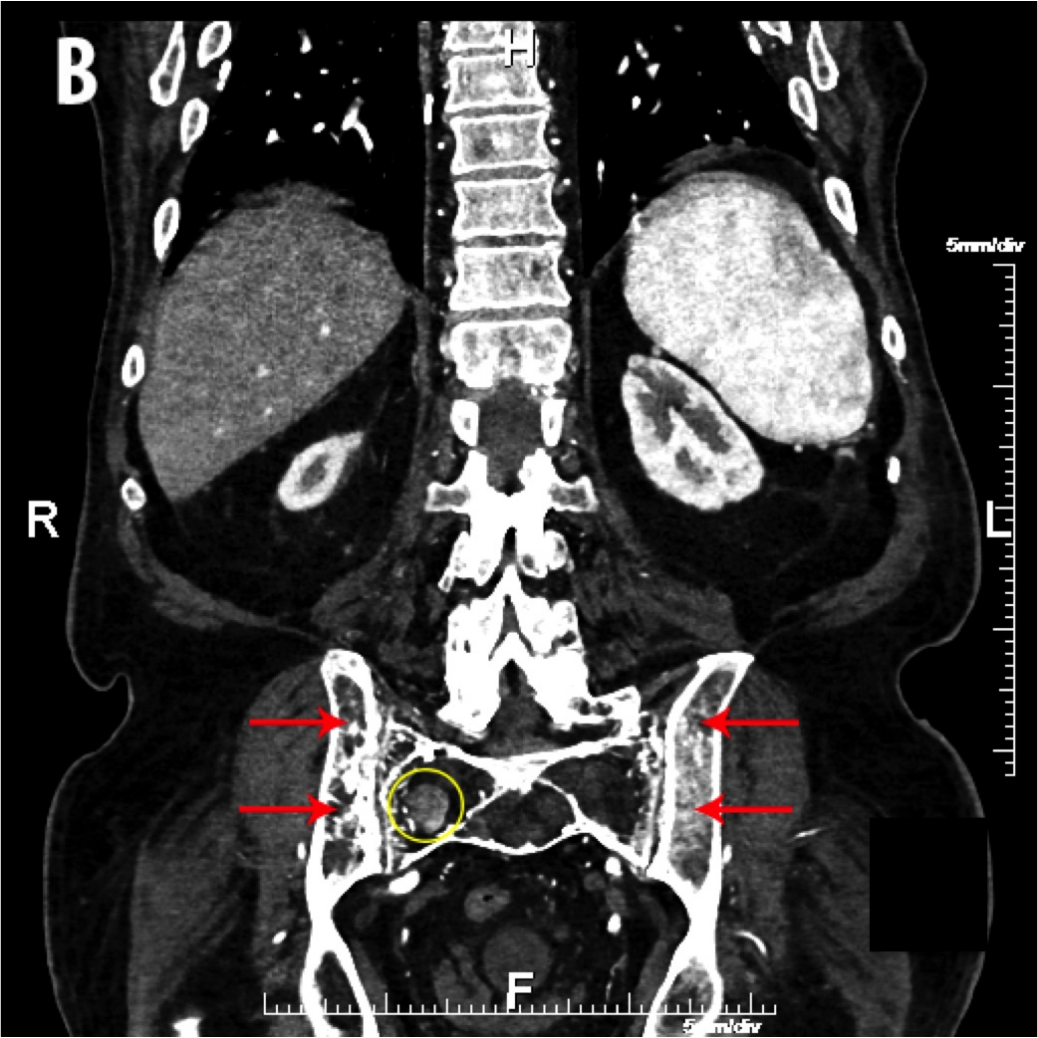
A Coronal CT of the Abdomen and Pelvis Indicating Metastatic Renal Cell Carcinoma in Pagetic Bone A coronal contrast-enhanced CT through the abdomen and pelvis: concomitant Paget’s disease (red arrows) and metastatic renal cell carcinoma (yellow circle) in the right sacrum.

Due to the severity of the abdominal pain, a computed tomography (CT) angiogram was performed to exclude mesenteric ischemia. A heterogenous, enhancing mass was identified in the lower pole of the right kidney with invasion of the right renal vein and inferior vena cava (IVC) (Figure 1). Osseous findings of coarse and thickened trabecula, cortical sclerosis, and enlargement were seen in the pelvis and sacrum, characteristic of Paget’s disease of the bone (Figure 2). Within the Paget’s disease of the upper right sacrum, there was a circumscribed, enhancing mass without calcification (Figure 3). A CT-guided biopsy of the right renal mass confirmed a diagnosis of renal cell carcinoma.

The patient opted to forgo biopsy of the mass in the right sacrum. The lack of typical osteoid matrix, aggressive periosteal reaction, and the lesion being centered in the medullary bone and not in the cortex indicated that this sacral mass likely represented a metastasis rather than a secondary osteosarcoma in Paget’s disease of the bone. Given the patient’s advanced age and comorbiditities, the patient and his family decided not to pursue further treatment.

## Discussion

Paget’s disease of the bone is an uncommon condition, which affects an estimated three percent of individuals over the age of 55. There is a slight (3:2) male preponderance [3-4]. Paget’s disease most commonly affects those of European descent. The disease is rarely reported among Asian and Scandinavian populations [1-4, 6]. Both genetic and environmental factors have been suggested to contribute to disease occurrence; however, the involvement of environmental factors is highly disputed [1, 6]. Paget’s disease of the bone is an autosomal dominant condition with variable penetrance and can have a familial or sporadic nature, with 12-40% of patients having a positive family history [1]. Histologically, Paget’s disease of the bone is typically characterized by the presence of large, supernucleated osteoclasts, an increased number of osteoblasts as well as the presence of bone remodeling, hypertrophy and an abnormal matrix structure at affected sites [1, 4, 6]. Abnormality of both the osteoclasts and osteoblasts results in a combination of increased bone resorption and formation. However, this newly formed bone is of poor quality, causing osteosclerosis and characteristic deposition of architecturally and lamellarly disorganized bone [1, 6]. Paget’s disease of the bone can present as localized monostotic (10-35% of cases) or polyostotic (65-90%) disease. In monostotic disease only one area is affected, whereas in polystotic disease multiple areas are affected. The axial skeleton, long bones, skull, spine, and pelvis are most commonly affected [1, 7-8].

Serum alkaline phosphatase (S-ALP) is a sensitive biochemical marker reflective of bone formation and thus is often used as a diagnostic measure. In Paget’s disease of the bone, a strong correlation between increased S-ALP levels and the extent of the disease has been found. However, S-ALP levels can be normal in early stages of monostotic disease and in some cases of polyostotic disease [1, 4, 6]. There are four cardinal radiographic features of Paget’s disease of the bone: advancing osteolysis, coarsening and thickening of bone trabeculae, subperiosteal cortical thickening, and osseus widening [4]. Different combinations of these features are often indicative of the phase of the disease. There can also be varying phases depending on the location of the disease. In long bones such as the femur and tibia, advancing osteolysis (seen as the 'blade of grass', 'cutting cone', or 'candle flame' sign) and focal bone sclerosis can be observed [1, 4]. Signs such as osteosclerosis and osseus enlargement are characteristic of the pagetic pelvis. However, occasionally the trabeculae can become obfuscated and produce a hazy ‘ground-glass’ or ‘washed out’ appearance within the area of radiolucency [4], similar to the appearance of the sacrum in our patient. In later stages of the disease, further osteosclerosis and fatigue fractures can be observed along with the cardinal signs [4].

Paget’s disease of the bone can also put the individual at an increased risk for bone neoplasms. The most common sites for malignant transformations are the femur, pelvis, and humerus [8]. Osteosarcoma develops in 0.2-1% of polyostotic patients. In cases of malignant transformation, osteosarcoma is the most common diagnosis (50-60% of cases). Rarely, malignant fibrous histiocytoma, fibrosarcoma, and chondrosarcoma can also be seen in Paget’s disease of the bone [3].

Metastatic disease to pagetic bone can also occur [3, 5]. This is hypothesized to occur because of the hypervascularity of pagetic bone during high turnover phases. This creates an optimal environment for metastatic disease [3, 5]. Breast, lung, and prostate carcinomas are the most common to metastasize to pagetic bone [9]. Renal cell carcinoma associated with Paget’s disease of the bone is very rare, with only one prior reported case [10].

## Conclusions

Although patients with Paget’s disease of the bone are at increased risk for primary bone neoplasms, such cases are rare, and metastatic disease to pagetic bone is extremely rare. We report a patient diagnosed with concomitant renal cell carcinoma and metastatic disease within Paget’s disease of the sacrum. Further research is needed to assess the true incidence of renal cell carcinoma associated with pagetic bone.

## References

[1] Al-Rashid M, Ramkumar DB, Raskin K, Schwab J, Hornicek FJ, Lozano-Calderon SA (2015). Paget disease of bone. Orthop Clin North Am.

[2] Cooper C, Dennison E, Schafheutle K, Kellingray S, Guyer P, Barker D (1999). Epidemiology of Paget's disease of bone. Bone.

[3] Lopez C, Thomas DV, Davies AM (2003). Neoplastic transformation and tumour-like lesions in Paget's disease of bone: a pictorial review. Eur Radiol.

[4] Theodorou DJ, Theodorou SJ, Kakitsubata Y (2011). Imaging of Paget disease of bone and its musculoskeletal complications: review. Am J Roentgenol.

[5] Fenton P, Resnick D (1991). Metastases to bone affected by Paget's disease. A report of three cases. Int Orthop.

[6] Siris ES (1998). Paget's disease of bone. J Bone Miner Res.

[7] White G, Rushbrook J (2013). Paget’s disease of bone. Orthop Trauma.

[8] Davie M, Davies M, Francis R, Fraser W, Hosking D, Tansley R (1999). Paget’s disease of bone: a review of 889 patients. Bone.

[9] Mundy GR (1997). Mechanisms of bone metastasis. Cancer.

[10] Burgener FA, Perry PE (1977). Solitary renal cell carcinoma metastasis in Paget's disease simulating sarcomatous degeneration. Am J Roentgenol.

